# COVID-19 in patients with thymic epithelial tumors with or without Good’s syndrome: a single-center retrospective study

**DOI:** 10.1186/s12885-024-12405-4

**Published:** 2024-06-19

**Authors:** Erica Pietroluongo, Annarita Peddio, Pietro De Placido, Marianna Tortora, Margaret Ottaviano, Monica Gelzo, Gustavo Cernera, Maria Foggia, Antonio Riccardo Buonomo, Biagio Pinchera, Emanuela Zappulo, Simona Mercinelli, Letizia Cattaneo, Alessia Sardanelli, Giulio Viceconte, Riccardo Scotto, Nicola Schiano Moriello, Alberto Servetto, Carmine De Angelis, Grazia Arpino, Giovannella Palmieri, Sabino De Placido, Roberto Bianco, Giuseppe Castaldo, Ivan Gentile, Mario Giuliano

**Affiliations:** 1 Department of Clinical Medicine and Surgery, University of Naples Federico II, Naples, Italy; 2 Department of Advanced Biomedical Sciences, University Federico II, Naples, Italy; 3 Department of Medical Oncology, Dana-Farber Cancer Institute, Boston, MA USA; 4 Regional Coordinating Center for Rare Tumors (CRCTR) of Campania Region at University Federico II, Naples, Italy; 5 Unit of Melanoma, Cancer Immunotherapy and Development Therapeutics, Istituto Nazionale Tumori IRCCS Fondazione G. Pascale, Napoli, 80131 Italy; 6 CEINGE, Biotecnologie Avanzate, Naples, Italy; 7 Department of Molecular Medicine and Medical Biotechnologies, University of Naples Federico II, Naples, Italy; 8 Department of Clinical Medicine and Surgery, Section of Infectious Diseases, University of Naples Federico II, Naples, Italy

**Keywords:** SARS-CoV-2, COVID-19, thymic epithelial tumors, Good’s syndrome

## Abstract

**Introduction:**

Thymic epithelial tumors (TETs) are rare neoplasms often associated with immune-related disorders. Patients with Good’s syndrome (GS), an adult-acquired TET-related immunodeficiency, are at a high risk of mortality due to infectious diseases. This study aims to examine COVID-19 occurrence and severity in TET patients, with or without GS.

**Methods:**

Clinical records of TET patients referred to the Regional Coordinating Center for Rare Tumors of Campania Region were retrospectively collected. During the observation period, elapsing from March 2020 to April 2023, the following data were collected: occurrence of SARS-CoV-2 infection; COVID-19 severity, according to the National Institute of Health (NIH) illness categories; COVID-19 treatment. COVID-19 occurrence and severity were assessed in the overall population and correlated with the presence of GS and/or other immune-related dysregulations.

**Results:**

Overall, 47 TET patients were included in the study; 27 of these (57.4%) had GS. All participants had received a full cycle of mRNA vaccine for SARS-CoV2., Thirty-one patients (66.0%) experienced COVID-19, of whom 18 (58.0%) had previously received a diagnosis of GS. No significant association of GS and/or other immune-related dysregulations with SARS-CoV-2 infection occurrence was detected (Fisher’s exact test *p* = 1 and *p* = 0.3587, respectively). Among patients with GS, 8 (45.0%) reported a COVID-19 severity score of ≥ 3; whereas, only 1 of the 13 patients without GS (7.7%) had a severity score of ≥ 3. The correlation between presence of GS and COVID-19 severity (score 1 or 2 vs. ≥ 3) was statistically significant (*p* = 0.0448). No statistically significant association between COVID-19 severity and other immune-related syndromes were found (*p* = 1). Of note, all the hospitalized patients for NIH 4 and 5 COVID-19 had GS.

**Conclusions:**

Our data suggest that TET patients, especially those with GS, require a careful multidisciplinary monitoring for SARS-CoV-2 infection, in order to establish tailored treatments and prophylactic protocols.

**Supplementary Information:**

The online version contains supplementary material available at 10.1186/s12885-024-12405-4.

## Introduction

Thymic epithelial tumors (TETs) are rare neoplasms, classified as thymoma (T) and thymic carcinoma (TC). Although they are the most common mediastinal malignancies, their incidence in the general population is less than 1% [1].

The clinical features and outcome of TETs are very heterogeneous, also due to several paraneoplastic syndromes that can be associated with these malignancies and impact patients’ survival and quality of life. These include neurological disorders, such as myasthenia gravis (MG) (incidence of 30–40%), and other less common immune system abnormalities [2].

Among these, Good’s Syndrome (GS) is particularly relevant. GS is an adult acquired immune-related immunodeficiency associated with TETs (6–11%), most commonly reported in patients affected by histotype A of thymoma [3]. The physiopathology of immunodeficiency in GS remains elusive. The possible explanations for the association between B-lymphopenia and GS may include the effect of cytokines, possibly secreted by bone marrow stromal cells, on growth and differentiation of thymic and B-cell precursors [4]. Moreover, studies of paraneoplastic phenomena in thymoma suggested that humoral and/or cell-mediated auto-immune responses may directly or indirectly inhibit B lymphopoiesis and immunoglobulin production by mature B cells [4, 5].

This syndrome is defined by the presence of hypogammaglobulinemia, B lymphopenia, CD4 T lymphopenia, abnormal CD4/CD8 ratio, and impaired T cell mitogenic responses associated with recurrent infections, due to encapsulated bacteria, fungi and viruses [3].

The link between thymic malignancies and changes in the immune system can be traced back to the thymus’ function in selecting and maturing T-cells. When neoplasms subvert the structure of the thymus, T cells can adopt autoreactive behaviour due to the lack of immune control in the thymic microenvironment. Although the origin of the B cell deficiency in these patients is unknown, a validated hypothesis is that a bone marrow alteration causes the arrest of B cell development, resulting in defects in antigen recognition and inability to fight pathogens [6–8].

GS and its related infections are major causes of death in TET patients [9], making them a remarkably vulnerable population, particularly during COVID-19 pandemic. Moreover, managing COVID-19 and its respiratory complications is challenging for cancer patients, particularly for those affected by mediastinal tumors, due to symptoms related to both cancer burden and prior or concurrent medical treatments. In this complex scenario, TET patients need close monitoring and require personalized multidisciplinary approaches for clinical management [10], although no clear indications in this field have been established due to the rarity of TETs associated with GS. Indeed, data on clinical management and outcomes of COVID-19 in TET patients have been available so far only from case reports.

Therefore, we conducted a retrospective study with the aim to evaluate occurrence, severity and multidisciplinary treatment of COVID-19 in a cohort of TET patients with or without GS and/or other immune-related dysregulations.

## Methods

This was a retrospective study conducted in a population of consecutive TET patients referred to the Regional Coordinating Center for Rare Tumors (CRCTR) of Campania Region (University of Naples Federico II). Inclusion criteria included age 18 years or older, histological diagnosis of TET, known disease status, diagnosis or not of Good’s syndrome, defined as previously described [11].

The observation period elapsed from March 2020 to April 2023.

The following data relative to SARS-CoV-2 acute infection and clinical management were collected:

- SARS-CoV-2 occurrence, before or after vaccine administration, determined using approved testing methods (PCR molecular test and rapid antigen-test with defined cut off index).
- Reinfection, defined as a new positive SARS-CoV-2 test occurring ≥ 90 days after resolution of the initial SARS-CoV-2 infection. To evaluate the duration of the swab positivity, the dates of the first positive and first negative SARS-CoV-2 tests were recorded.
- Clinical severity of COVID-19, was evaluated according to the severity of illness categories of the National Institutes of Health (NIH) [12], and associated to a score from 1 to 5 as follows:
  1. *Asymptomatic or pre-symptomatic infection*.
  2. *Mild illness* (patients show mild symptoms such as fever, coughing, altered taste, malaise, headache, and myalgia. No dyspnea or radiologically detectable signs are reported).
  3. *Moderate illness*, defined by a peripheral oxygen saturation level (SpO2) of 94% or higher in ambient air, as detected by a pulse oximeter, with clinical or radiological evidence of pneumonia.
  4. *Severe disease*, characterized by a SpO2 lower than 94% in at least one measurement.
  5. *Critical illness*, associated with respiratory failure, septic shock and/or organ failure (respiratory rate higher than 30 breaths per minute, SpO2 in arterial blood lower than 93%, PaO2/FiO2 lower than 300, and/or pulmonary infiltrates present in more than 50% of the lung parenchyma).

Vaccine prophylaxis was performed by administering first, second, and booster dose of mRNA BNT162b2 vaccine (Pfizer-BioNTech) [13]. Patients still serologically negative for anti-SARS-CoV-2 antibodies after completion of the full mRNA vaccine cycle (first and second dose) received the monoclonal antibody combination tixagevimab/cilgavimab, according to *Agenzia Italiana del Farmaco* (AIFA) recommendations [14].

Treatment for acute COVID-19, including use of antiviral therapy (remdesivir, molnupinavir, nimatrelvir/ritonavir, ) or monoclonal antibodies (mAbs) (casirivimab/imdevimab, sotrovimab) was administered according to AIFA recommendations [15].

COVID-19-related deaths were defined as events occurring during the course of acute SARS-Cov-2 infection, in presence of positive swab test (PCR molecular test and/or rapid antigen-test).

Disease status (NED/ED), as well as presence of GS, MG and any autoimmune disease (AD) were correlated with severity of COVID 19 using as cut-off the severity score of 2 (i.e., 1 or 2 vs. ≥ 3). In case of re-infection occurring in the same patient, the highest observed severity score was considered.

Patient characteristics were summarized using media, medians, and percentages as appropriate. For categorical data Fisher’s exact tests were used. Mann-Whitney U test was used for non-parametric distribution. A multivariable logistic regression model was built to further validate significant univariate associations between variables. Odds Ratio (OR) and its relative 95% confidence interval were reported. Statistical significance was considered as a two-tailed *p* < 0.05. Statistical Analyses were performed using GraphPad Prism 9.0 software (GraphPad Software Inc, La Jolla Ca).

## Results

### Baseline characteristics

Overall, 47 TET patients were included in this study. Patient and tumor characteristics are shown in Table 1.

**Table 1:** Patient characteristics

Characteristics	Number of patients (%)
ECOG PS	
0 1 Age, median (range)	34 (72.3%) 13 (27.7%) 57 (33–76)
Sex	
Male Female	20 (42.6%) 27 (57.4%)
Histological Type	
Thymic Hyperplasia Thymoma A AB B1 B2 B1-B2 B2-B3 B3 Not otherwise specified Thymic carcinoma Thymic neuroendocrine tumor	0 5 (10.6%) 8 (17%) 5 (10.6%) 9 (19.1%) 0 3 (6.4%) 7 (14.9%) 1 (2.1%) 8 (17%) 1 (2.1%)
Radiological Stage of Disease according to TNM	
I II III IVA IVB	11 (23.4%) 4 (8.5%) 5 (10.6%) 14 (29.8%) 13 (27.7%)

ECOG PS: Eastern Cooperative Oncology Group Performance Status

Thirty-eight patients (80.9%) were diagnosed with thymoma, 8 (17%) with thymic carcinoma, and 1 (2.1%) with thymic neuroendocrine tumor. At the time of study initiation, 22 patients (46.8%) were in follow-up with no evidence of disease (NED), whereas the remaining 25 patients (53.2%) had evidence of disease (ED) and were receiving systemic treatment (Table 2). Twenty-seven patients suffered from GS (57.4%).

**Table 2:** Systemic treatment administered in TET patients with evidence of disease (ED)

Anti-tumor treatment	Total *N* (%)	TET with GS	TET without GS
Etoposide-based chemotherapy	14 (56%)	10 (40%)	4 (16%)
Octreotide-LAR	9 (36%)	4 (16%)	5 (20%)
Platinum based- chemotherapy	2 (8%)	1 (4%)	1 (4%)
Total	25 (100%)	15 (60%)	10 (40%)

*Etoposide-based chemotherapy: 9 single-agent oral low-dose etoposide; 3 pembrolizumab + oral low-dose etoposide; 2 capecitabine + oral low-dose etoposide*

Other autoimmune disorders (AD) were detected in 24 patients (51.1%), with MG being the most frequent autoimmune disorder, diagnosed in 15 patients (31.9%) (Table 3).

**Table 3:** Immune-related disorders

Immune-related disorders	Number of patients (%)
None	20 (42.6)
Good’s Syndrome (GS)	27 (57.4)
Other immune-related disorders Myasthenia Gravis (MG)* Lambert-Eton Pure Red Cell Aplasia Isaacs Syndrome Rheumatoid Arthritis Thyroiditis Polymyositis	24 (51.1) 15 (31.9) 1 (2.1) 4 (8.5) 1 (2.1) 1 (2.1) 1 (2.1) 1 (2.1)

*Eight patients have concomitant MG and GS

All participants had received a full cycle of mRNA vaccine for SARS-CoV2, which was administered within the observation period. Six patients (7.8%) received also anti-SARS-CoV-2 prophylaxis with the monoclonal antibody combination tixagevimab/cilgavimab.

### Occurrence and severity of COVID-19

Overall, 31 patients (66.0%) experienced SARS-CoV-2 infection during the observation period. Of these, 18 (58%) had GS. Four patients with GS and 6 patients without GS had confirmed re-infections during the observation period. The occurrence of first SARS-CoV-2 infection was reported in 12 cases (39%) before the administration of the first dose of mRNA vaccine, whereas in the remaining cases first occurrence of infection was reported after the second dose of vaccine or after the booster dose.

No statistically significant correlation was found between the occurrence of SARS-CoV-2 infection and disease status (NED vs. ED) (Fisher’s exact test *p* = 0.2158). Moreover, no significant correlation between the presence of either GS, AD or MG and the occurrence of SARS-CoV-2 infection was detected (Fisher’s exact test *p* = 1, *p* = 0.3587, and *p* = 1, respectively).

Among the 31 patients with documented SARS-CoV-2 infection, the median score for COVID-19 severity according to NIH classification was 2.

The severity of COVID-19 (severity score 1 or 2 vs. ≥ 3) was not significantly associated with timing of infection (i.e., infection occurring before vs. after vaccine administration) (Fisher’s exact test *p* = 1). Moreover, no statistically significant correlations of COVID-19 severity with disease status (NED vs. ED) (Fisher’s exact test *p* = 0.0732) and with presence of MG or AD (Fisher’s exact test *p* = 0.4521 and *p* = 1 and, respectively) were found.

**Fig. 1:**
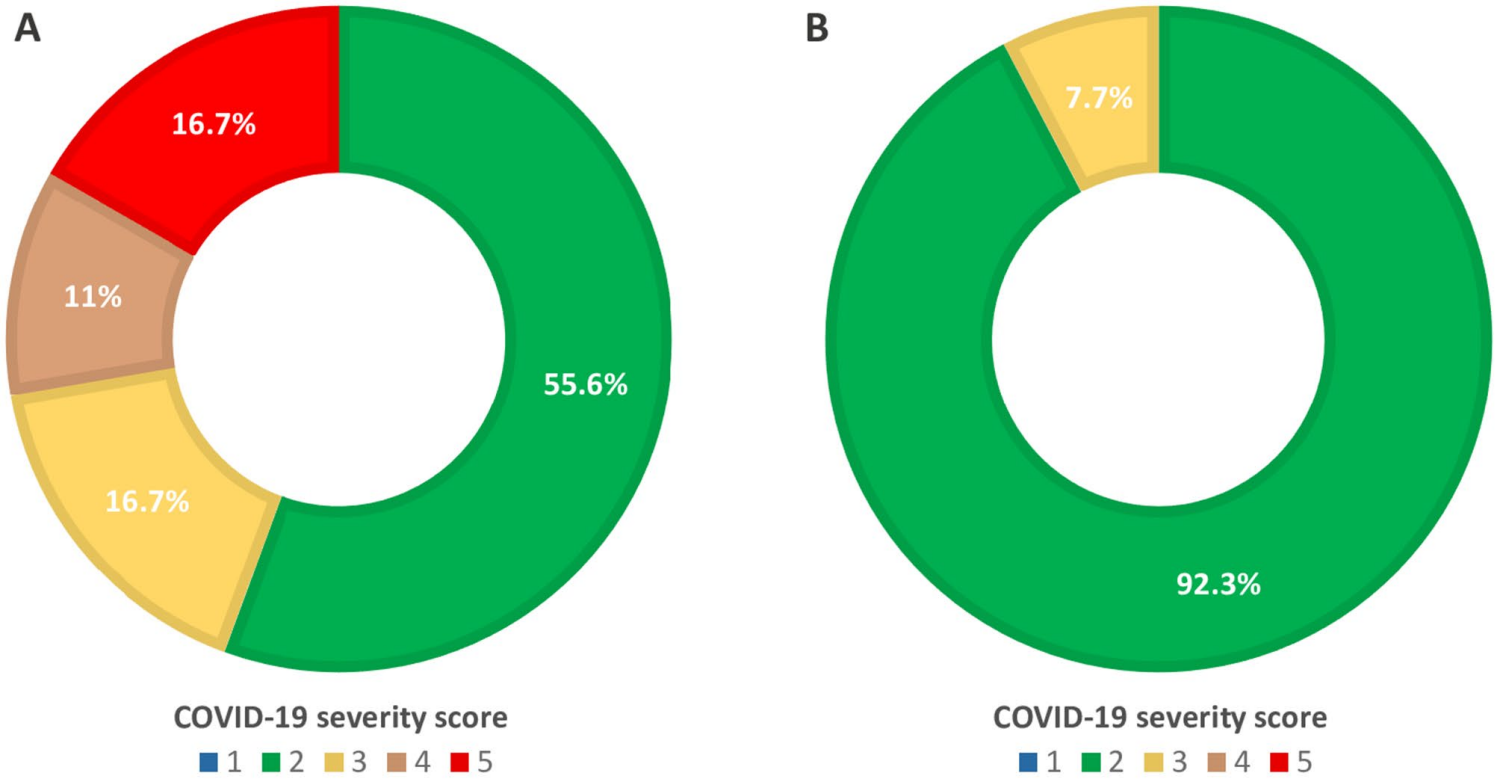
COVID-19 severity score in patients with GS (1 A) and without GS (1 B).

In the group of patients with GS, median severity score was 3. In detail, 10 cases (55.6%) reported a severity score of 2, 3 cases (16.7%) a score of 3, 2 cases (11%) a score of 4, and 3 cases (16.7%) a score of 5 (Fig. 1A).

The hospitalization rate among patients with GS who experienced COVID-19 was 27.7%. In 5 cases, oxygen support was needed. The median duration of hospitalization was 28 days (range 14–150). The median overall duration of SARS-CoV-2 positivity was 19.5 days (range 11–344).

Among the 13 TET patients without GS, 12 (92.3%) had a severity score of 2, and 1 (7.7%) a score of 3 (Fig. 1B). No patient without GS required oxygen support or hospitalization. The median duration of SARS-CoV-2 infection in this subgroup was 17 days (range 9–58).

A statistically significant correlation was found between presence of GS and COVID-19 severity (score 1 or 2 vs. ≥ 3) (Fisher’s exact test *p* = 0.0448). This association retained its significance when adjusted for age, as well as cardiovascular and metabolic comorbidities (*p* = 0.040; OR: 12.51; 95%: CI: 1.12 to 139.66) (Supplementary Table 1). On the other hand, no statistically significant correlation was found between median duration of SARS-CoV-2 infection and presence of GS (17 vs. 19.5) (Mann Whitney test *p* = 0.2706).

### Antiviral therapy and patient outcome

Among the 18 patients with GS who experienced COVID-19, 6 received direct antiviral therapy. Of these, 3 patients were treated with intravenous (iv) remdesivir during their hospitalization due to severe COVID-19; the remaining 3 patients received either oral molnupinavir (1 patient) or oral nimatrelvir/ritonavir (2 patients) (Table 4). In addition, 4 patients received iv monoclonal antibody therapy for COVID-19, with either casirivimab + imdevimab (3 patients) or sotrovimab (1 patient). Three patients received both antiviral therapy and monoclonal antibody during their hospitalization (Table 4).

**Table 4:** COVID-19 severity in TET with and without GS, therapy, and hospitalization rate

Group	Total Patients	Cases of COVID-19	Cases of severe COVID-19 *	Antiviral Therapy	Monoclonal Antibody Therapy	Median Severity Score	Hospitalization Rate
Patients with GS	27	18 (66.6%)	8** (45%)	3 Remdesivir, 1 Molnupinavir, 2Nimatrelvir/Ritonavir	3 Casirivimab + Imdevimab 1 Sotrovimab	3	27.7%
Patients without GS	20	13 (65.5%)	1 (8%)	2 Molnupinavir, 2Nimatrelvir/Ritonavir	None	2	0%

*Severe COVID-19 cases represent the number and percentage of COVID-19-positive patients whose disease severity was moderate or higher (NIH severity score ≥ 3)

** 4 patients received both antiviral therapy and monoclonal antibody

Among the 13 TET patients without GS who experienced COVID 19, 4 received oral antiviral therapy either with molnupinavir (2 patients) or with nimatrelvir/ritonavir (2 patients) (Table 4).

No statistically significant correlation was found between antiviral therapy administration and COVID-19 severity (*p* = 0.1277).

Overall, no patient died during acute SARS-CoV-2 infection. However, 2 patients with GS died respectively 2 and 4 weeks after the resolution of the viral infection, due to bacterial lung infections. Notably, 1 of these patients had also received monoclonal treatment (tixagevimab/ cilgavimab) as prophylaxis 4 weeks before testing positive for SARS-CoV-2 infection.

## Discussion

Cancer patients have been significantly affected by COVID-19. The pandemic has caused a major upheaval in healthcare, leading to delays in medical treatments, development of triage strategies [16] and reduced accessibility to necessary services for these fragile individuals. TET patients are particularly vulnerable, being affected by both intra-thoracic tumors and immune syndromes that can complicate their treatments and worsen their prognosis. Among the aforesaid syndromes, the rarest and least known is GS, which also represents the major cause of death in these patients [17].

SARS-Cov-2 vaccine have dramatically contributed to reduce COVID 19 occurrence, severity and mortality in both healthy individual and cancer patients [18]. A recent study carried out by our group showed safety of mRNA vaccine in TET patients, with no reported cases of worsening autoimmunity or other complications. However, the same study showed that among the 39 TET patients who underwent the full mRNA vaccine cycle, approximately half did not achieve seroconversion [19]. In a subsequent analysis evaluating both humoral and cell-mediated immunity following the administration of the booster dose, we identified a subgroup of patients who failed to achieve seroconversion despite completing the full vaccine cycle, although they exhibited cell-mediated immunity. Nevertheless, a limited proportion of patients did not develop either humoral or cellular responses [20].

In this complex scenario, we sought to evaluate clinical outcome and describe multidisciplinary treatment of TET patients experiencing COVID-19 before or after receiving mRNA vaccine. Above all, we investigated the potential impact of GS on COVID 19 severity.

Our study showed no increased risk of SARS-Cov-2 occurrence in TET patients with GS as compared to those without GS. It could be hypothesized that our findings may be due, at least in part, to careful prevention adopted by particularly fragile TET patients (i.e., careful use of masks and other medical safety measures, avoidance of contact with symptomatic individuals) as well as to the high attack rate of this virus. However, the limited sample size and the retrospective nature of our study cannot allow us to draw any conclusion on this issue, which can be clarified only by larger population-based studies.

Interestingly, though, TET patients with GS showed more severe clinical features of COVID-19, as compared to those without GS, reporting a hospitalization rate of 27.7%, vs. 0%, regardless of a previous history of SARS-CoV-2 infection acquired before or after mRNA vaccination. Multivariate analysis confirmed the association between presence of GS and COVID-19 severity, independently from other known risk factors, such as age, as well as cardiovascular and metabolic comorbidities. Moreover, the severity of infection in our GS cohort appeared to be higher than that reported in the general population (global average hospitalization rate and severity rate of 23.36 and 8.53 per 1000 infections, respectively) [21].

Furthermore, our findings are consistent with the results of other studies assessing the impact of COVID-19 in immunocompromised patients. Fung and Babik performed an extensive literature review of COVID-19 in patients with cancer (including hematologic malignancies), solid-organ transplant recipients, individuals with HIV, and those receiving immunomodulatory therapies for autoimmune disorders (such as antirheumatic drugs, biologics for inflammatory bowel disease, among others). The study showed that cancer patients and transplant recipients were at higher risk of severe COVID-19 and death. No significant evidence of major risks was found among other immunocompromised patient subgroups [22]. Additionally, Seth et al., confirmed the finding that cancer patients are more vulnerable to severe acute SARS-CoV-2 infection and have a poorer prognosis as compared to the general population. In addition, immunosuppressive antitumor treatment did not affect COVID-19 fatality rates, and there was no correlation between histology and clinical outcomes [23]. Similarly, we believe that systemic anti-cancer treatments administered at the time of SARS-Cov-2 infection did not affect COVID-19 severity and outcome in our cohort of patients. As matter of fact, patients with ED undergoing systemic treatment did not show a significantly higher COVID 19 severity as compared with NED patients who were in follow up at the time of SARS-Cov-2 infection. On the other hand, the profound immunodeficiency depicted by B lymphopenia and abnormal CD4/CD8 ratio present in TET patients with GS, could substantially impact severity and disease course of COVID-19. Consistently, previous studies in patients with clinical conditions linked to B lymphopenia, such as X-linked agammaglobulinemia (XLA) and common variable immunodeficiency (CVID), have revealed higher risk of severe outcome during the course of COVID-19 [24–26].

Definitely, COVID-19 pandemic has provided a valuable model and a further incentive to achieve a more comprehensive understanding of physiopathology of GS that may help to better dissect the clinical features of this complex and poorly known immune dysregulation to in order to establish tailored treatments and prophylaxis protocols for acute SARS-CoV-2 infection.

## Conclusion

To the best of our knowledge, this is the first study evaluating the course of COVID-19 in patients affected by TET. Despite its small sample size and retrospective design, we believe that our study could have meaningful clinical implications as it suggests that TET patients, particularly those with GS, have a higher rate of hospitalization and risk of severe outcome for COVID-19, regardless of active anticancer treatments. For these individuals deemed especially vulnerable, the initiation of antiviral therapy upon a positive COVID-19 test result is recommended, along with sustained post-recovery monitoring.

Our findings may also prove useful in the multidisciplinary management of other infectious diseases, which are major causes of death in TET patients, especially in those affected by GS.

### Electronic supplementary material

Below is the link to the electronic supplementary material.

Supplementary Material 1

## Data Availability

All clinical data analysed in this study are detailed in electronic medical records.

## Abbreviations

SARS-CoV-2: severe acute respiratory syndrome coronavirus 2
TET: thymic epithelial tumors
GS: Good’s syndrome
T: thymoma
TC: thymic carcinoma
