# Supplementary material for: COVID-19 in patients with thymic epithelial tumors with or without Good’s syndrome: a single-center retrospective study
Source: BMC Cancer. 2024 Jun 19;24:748. doi: 10.1186/s12885-024-12405-4 (PMC11188232; doi:10.1186/s12885-024-12405-4)
Supplement: Supplementary file 1 — Supplementary Material 1 [file 12885_2024_12405_MOESM1_ESM.docx]

**Supplementary Table 1**. Multivariate Analysis of COVID-19 severity in TET patients

| **Variable** | **Odds Ratio** | **95% Confidence Interval** | **P value** |
| --- | --- | --- | --- |
| **Presence of GS** | 12.5 | 1.25-139.16 | **0.040*** |
| **Cardiovascular comorbidities^** | 0.41 | 0.05-3.05 | 0.385 |
| **Metabolic comorbidities^§^** | 2.72 | 0.26-27.63 | 0.398 |
| **Age**  **(≤ 65 vs > 65 years)** | 2.71 | 0.19-37.86 | 0.458 |

**Statistically significant (< 0.05).*

*^Cardiovascular comorbidities: story of coronary heart disease, arrhythmias, arterial hypertension.*

*^§^Metabolic comorbidities:* *dyslipidemia, diabetes.*
